# An evaluation system for scientific journals

**DOI:** 10.1038/s44319-025-00649-5

**Published:** 2025-12-08

**Authors:** Diethard Tautz, Paul B Rainey

**Affiliations:** Max-Planck Institute for Evolutionary Biology, Plön, Germany

**Keywords:** Careers, Science Policy & Publishing

## Abstract

Scientific journals disseminate research findings and serve as a currency for measuring the reputation of scientists. Given their importance for science and for scientists’ careers, we argue that there is a need to evaluate journals.

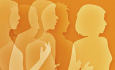

The primary function of scientific journals is to disseminate research findings, controlled by the community of scientists working in the field. Over time, however, journals have acquired a second, equally important role: serving as a currency for measuring the reputation of scientists (Trueblood et al, 2025; Petersen et al, 2014; Reinhart and Schendzielorz, 2024). These two functions have become inseparably linked, as researchers face constant evaluation, applied to institutions, research infrastructures, consortia, individual scientists, and grant applications, nearly all of which rely heavily on publications in scientific journals (Rainey et al, 2025a). Yet, paradoxically, there is no formal mechanism for evaluating the journals themselves. We therefore argue that there is a need to establish evaluation procedures for scientific journals.

Currently, the perceived value of a journal largely rests on an ad hoc metric: its impact factor (IF). This measure is based on the average number of citations articles receive over a given time period, usually 2 years. Superficially, this resembles how stock market values are determined through the activities of buyers—in the case of journals, it is readers who find an article sufficiently relevant to cite it in their own manuscript. However, there are two key differences. First, unlike buying assets on financial markets, citing articles cost nothing—making a free resource the basis of a value-generating system. Second, while stock market values rely to a large extend on the assessment by professional analysts, scientific publishing lacks an equivalent cadre of experts to evaluate journals’ quality and performance.

This situation has encouraged activities by publishers to manipulate the IF (Siler and Larivière, 2022). For instance, editors prefer articles that are likely to draw many citations in a short period, that is, emphasizing fashionable scientific trends over ground-breaking novelty that could take more than 2 years to demonstrate its real value. Further, since a few highly cited articles can disproportionately raise a journal’s score, editors may strategically publish broadly useful—and citable—tools, databases or methods, often updating them every 2 years to sustain the IF. An even more troubling development is the rise of citation-padding, particularly in the context of predatory publishing, where real or fake articles cite other—often irrelevant—articles from authors who have paid for these citations, with consequences on the IF of the journal.

The scientific community is well aware of these problems. Many funding agencies now discourage reliance on IFs and the number of papers in evaluation, urging committees instead to assess the actual content of an applicant’s publications. For instance, an evaluation committee should read only the top five publications of a person applying for a career position. In practice, however, this is not scalable. There can be hundreds of applicants, which would keep the committee busy for many weeks with just reading articles. Similarly, panels reviewing grant proposals cannot realistically scrutinize both the applications and the associated publications. Surrogate metrics remain therefore unavoidable, at least for shortlisting.

We are therefore in a situation where committees and administrators tend to fall back to an IF-based judgment, although it is well understood how poor it is in its essence. As a metric, it seems so deceptively simple and quantifiable. This makes it particularly attractive to administrators of science who, by necessity, do not have much chance to develop their own judgment of the inherent quality of the diversity of published works that they have to deal with when making decisions about funding allocations in their institutions.

An alternative is to base judgments on the general reputation of journals. Ideally, experts form opinions about a journal over time, based on their selectivity, the rigor of peer review, and important articles that were published there. But with the rapid growth of scientific outputs, the number of journals has exploded, and disciplinary communities have become fragmented. A reputation system based on personal experiences therefore also reaches its limits.

Historically, society journals served as reliable anchors of such reputational assessments (Rainey et al, 2025b). Owned and governed by learned societies, they were accountable to their research communities and not to shareholders, and revenues were typically reinvested into conferences, training, and early-career support. Their editorial boards upheld quality through scholarly judgment rather than market visibility. The collective expertise and commitment of this community give a journal its distinctive character and enduring value. Yet, even within this group, pressures to compete in the IF economy have eroded these values. Some society journals continue to exemplify transparency and community service; others have drifted toward the same revenue-driven selectivity as commercial publishers. This divergence illustrates both the strengths and vulnerabilities of reputation as a guide to journal quality.

These tensions point to a broader weakness of reputation as a currency of value. Reputational assessments are inherently unstable, which affects in particular younger researchers whose career prospects hinge on them. Journal reputations can rise or fall rapidly: new titles may build credibility only to decline once publishers prioritize revenue over quality, while experiments with new publishing models can devalue authors’ earlier work despite unchanged scientific merit. In such a volatile environment, researchers gravitate toward ‘safe’ journals associated with prestigious brands. This in turn amplifies market concentration: subsidiary journals gain authority by association with reputable titles even when their editorial standards are untested. In financial terms, reputation can behave less like equity grounded in intrinsic worth than like a speculative asset, detached from real scientific value.

Neither IFs nor reputation-based judgments adequately reflect the rigorous standards applied to individual publications. The gold standard remains peer review, where experts evaluate content directly. Despite its limitations, peer review is still the most effective mechanism for self-regulation in science. It follows that journals themselves should also undergo peer review, much like how stock analysts apply systematic criteria to assess value.

However, calls to extend peer review to journals face a practical barrier: reviewer fatigue. Competent peer review is already stretched thin, largely because it relies on unpaid labor that is considered part of scientists’ professional duties. As a free resource, it is therefore subject to overuse. Expanding it further risks exacerbating the problem.

To move forward, it is necessary to develop new mechanisms for evaluating journals that do not overburden the peer-review system. Ideally, public payments for journal services, such as Open-Access fees, should be conditional upon journal accreditation against transparent, enforceable standards (Timmis et al, 2025). Criteria could include: a well-constituted editorial board reflecting diverse expertise and career stages with transparent governance; a documented, transparent and auditable peer-review processes with clear acceptance and rejection criteria following the standards of scholar-led publishing; public disclosure of cost calculation, waiver policies and editorial conflicts of interest; minimum benchmarks for time-to-decision and review quality; and commitment to a code of conduct that rejects predatory practices.

This could best be achieved by clearinghouses for journal evaluation and certification that establish accreditation and reputation criteria based on transparent, standardized benchmarks subject to continuous monitoring. A coalition of national funders, indexing services, and learned societies could set up discipline-specific clearinghouses (Timmis et al, 2025). Alternatively, they could be run on a commercial basis similar to rating agencies for financial markets. The IF metric is already provided by a commercial supplier, but without appropriate scientific control and unclear revenue-generating mechanisms. Commercial clearinghouses that operate on a set of criteria that are established by the scientific community could sell their services to publishers, funders, and science administrators in an open business model. Such services should be paid for—if they work openly and only for scientific purposes since they would be at the heart of the value-generating system for scientific output.

Such mechanisms would provide a more sustainable basis for uniting the functions of scientific journals: to disseminate reliable research results and to serve as a currency for measuring the achievements of scientists. It could also immunize against the predatory and fraudulent practices that are currently threatening the scientific publication system (Richardson et al, 2025; Sabel et al, 2025). And it would generate a mechanism for grant systems that could finance journals according to the Diamond open-access standards as a basic infrastructure for science (e.g. Tautz et al, 2025).

## Supplementary information

Peer Review File
