# Supplementary material for: An evaluation system for scientific journals
Source: EMBO Rep. 2025 Dec 8;27(1):3–4. doi: 10.1038/s44319-025-00649-5 (PMC12795802; doi:10.1038/s44319-025-00649-5)
Supplement: Supplementary file 1 — Peer Review File [file 44319_2025_649_MOESM1_ESM.pdf]

EMBO reports

An Evaluation System for Scientific Journals

Diethard Tautz and Paul Rainey

Corresponding author(s): Diethard Tautz (tautz@evolbio.mpg.de)

Review Timeline:

Submission Date:	27th Oct 25
Accepted:	6th Nov 25

Editor: Holger Breithaupt

Transaction Report: This article was editorially reviewed.
